# On the Road to Safety: Examining Children’s Cycling Skills and Physical Activity Levels

**DOI:** 10.3390/children11121556

**Published:** 2024-12-22

**Authors:** Juliane Stark, Michael Meschik

**Affiliations:** University of Natural Resources and Life Sciences Vienna, Department of Landscape, Spatial and Infrastructure Sciences, Institute of Transport Studies, Peter Jordan St. 82, 1190 Vienna, Austria; michael.meschik@boku.ac.at

**Keywords:** cycling skills, children, parental attitudes, active mobility, physical activity

## Abstract

Background/Objectives: Studies indicate a decline in children’s physical activity (PA) levels, active mobility, and psychomotor skills, reflected in poorer cycling abilities. These trends are worrying given the rising prevalence of childhood obesity and sedentary lifestyles. This study assessed cycling skills among primary school children in Lower Austria, comparing self-perceptions with objective assessments, and explores the relationship between cycling skills, PA levels, and school performance, as well as the impact of cycling training on skill development. Methods: A multi-level approach was employed, including cycling tests and interviews with children, parents, and teachers. Children’s cycling skills were evaluated through objective assessments, while parents and children provided self-assessments. Interviews explored children’s preferences, physical activity and travel habits, and school performance. Results: Overall, the children’s cycling skills were rated as good, though turning left, slalom, and emergency braking proved particularly challenging. Significant discrepancies were identified between objective assessments and self-perceptions, with many children—especially girls—overestimating their abilities. Despite some tendencies, no significant associations were found between PA levels or school performance and cycling skills. However, cycling training was significantly associated with improvements in cycling skills. Conclusions: In light of children’s strong preference for incorporating more cycling into their daily travel patterns, the findings underscore the importance of policies promoting safer school environments and encouraging parental support for cycling. More targeted cycling training programs are needed to further enhance children’s skills. It is also important to address the tendency of parents to overestimate their children’s cycling abilities. These measures could help foster greater use of bicycles for school commutes and improve children’s cycling competence and overall health outcomes.

## 1. Introduction

Literature highlights the concerning levels of physical inactivity among children and adolescents across Europe and North America [1,2,3,4]. For instance, in Austria, less than 18% of pupils aged 11 to 17 meet the World Health Organization’s (WHO) recommendations of 1 h of physical activity (PA) of moderate or strenuous exercise every day [5]. On average, in 2021/2022, girls meet these recommendations on only 3.8 days a week and boys on 4.5 days per week [6]. These low levels of physical activity correlate with worsening health and increasing obesity levels [6,7,8]. Moreover, decreasing activity levels adversely affect cognitive skills and psychological aspects such as concentration, confidence, self-esteem, and stress levels [9]. The negative development of decreasing physical activity is also reflected in the change in mobility patterns. Active mobility (AM), in addition to exercise (such as organized training), can be seen as an integral part of physical activity. However, the prevalence of car usage has led to generations becoming heavily reliant on automobiles, with parents and children increasingly opting for car travel even on short trips [10]. Trends in Austria, as observed through national household travel surveys from 1995 to 2013/2014, confirm this shift towards motorized modes, with a significant increase in car use, a strong decline in walking, and a rise in bicycle usage among the general population [11]. Notably, while the average daily distance cycled per person in Austria has doubled over the last two decades, these trends are not reflected among children: Detailed analyses from Austrian mobility surveys reveal a significant decrease in pupils’ bicycle trips to school, from 15% to 9%, with a corresponding decrease of 59% in total kilometers cycled to school [12]. Similarly, walking trips by pupils have decreased dramatically, while children are increasingly being driven to school [11,12]. Limited data are available for primary school children, but recent studies indicate a reliance on car travel, even for short distances, despite opportunities for active modes such as cycling and walking [13].

Furthermore, reduced independent mobility among children has been observed, especially for non-school destinations [14,15,16], with implications for their social interactions and physical activity levels [17,18,19]; further deterioration is to be expected [20]. Studies have shown that the decline in psychomotor capabilities among children, including bicycling skills, can be attributed to decreasing levels of active mobility and exercise [21]. Concerns about traffic dangers have led to parental constraints on children’s independent mobility, contributing to increased reliance on parental transport and reduced physical activity levels [22,23].

Experts attribute the development of children’s psychomotor capabilities primarily to their age, recommending cycle training as a preparation for on-street cycling [24,25,26]. Legislation varies regarding the age at which children are allowed to cycle independently on the streets. In Austria, the Road Traffic Regulations (StVO) allow children to ride a bicycle unaccompanied from the age of 12 years. However, Austrian traffic laws allow children to voluntarily take a “cycling exam” from the age of 9, often organized within primary schools (typically in the 4th grade) [27]. If they pass the test, they receive a “cycling pass”. Younger children have to use the sidewalks on children’s bicycles (up to 300 mm rim diameter), scooters, or other vehicle-like toys; they must also be accompanied by a person at least 16 years old, who is not allowed to use the sidewalks [27]. In contrast, German children are permitted to cycle on sidewalks independently without a minimum age limit and can cycle on public roads alone starting at age 8 [28]. Since December 2016, the accompanying persons of children under the age of 8 are also allowed to cycle on the sidewalk. The revised legislation also allows children to cycle unaccompanied on separate cycle paths. In Switzerland, this independence is granted from the age of 6 [28]. These different legal permissions reflect diverse approaches to a contentious issue: the balance between training children to be safe road users and improving traffic conditions to accommodate inexperienced young road users.

Passing the Austrian voluntary cycling exam requires a “certain level of physical and cognitive ability” [27], although the criteria for assessing this ability are not explicitly defined. The exam comprises both theoretical and practical components and is typically administered by the police in collaboration with schools [29]. In the theoretical segment, children must demonstrate knowledge of bicycle components, road markings, and traffic signs through a multiple-choice test consisting of 15 questions, with a minimum passing threshold of 80% correct answers. The practical portion involves executing tasks such as making left turns, cycling one-handed, and adhering to priority rules during maneuvers [30]. However, there is no standardized nationwide protocol for designing or evaluating practical tasks, leading to variations in assessment methods across regions. In recent years, it has become increasingly common to hear of children failing their cycling test [31,32,33]. There are no precise statistics on this. However, it is striking that the vast majority of children in rural areas pass the test—here the bicycle seems to be more important for becoming independent of parental transport. There is a wide range: In Eferding (a town in Upper Austria), all pupils passed the test in 2023—at least at the second attempt—while in Wels (a town in Upper Austria), one in five also failed at the second attempt [32].

This study endeavor seeks to enhance our understanding of the current state of cycling proficiency among primary school children and its relationship with physical activity levels. Furthermore, we assess the immediate impact of cycle training on the cycling abilities of 3rd and 4th graders in schools in the federal state of Lower Austria, prior to their participation in the voluntary cycling exam. A special aspect of the study is that the perspectives of the children, parents, and teachers were included. The study aims to test several hypotheses: firstly, whether children’s and parents’ perceptions of cycling skills are consistent with an objective assessment; secondly, whether cycling training improves cycling skills; and thirdly, whether physically active pupils show better cycling skills and academic performance.

## 2. Materials and Methods

### 2.1. Study Design

The primary objective of this study was to gather comprehensive information through a mixed-methods approach, combining quantitative and qualitative data collection techniques. Data were gathered through multiple sources, including (i) self-reports from primary school children, (ii) objective observations during cycling skill tests conducted before and after a 1 h cycling training session, (iii) interviews with parents, and (iv) interviews with teachers.

The sample was drawn from primary schools in Lower Austria that had registered for cycling workshops. These schools were invited to participate in the study, which took place between May and October 2016. Ultimately, five schools agreed to partake in additional surveys. Children in the 3rd and 4th grades were selected for the study, as the “voluntary cycling exam” according to Austrian traffic legislation is typically undertaken in this age group. At this stage, children’s motor skills and cognitive abilities, such as balance, braking, steering, maintaining track, and concentration, are expected to be sufficiently developed to ride a bicycle independently [24,25,34,35].

A total of 152 children (aged 8–10 years) from the five participating schools took part in the survey, with 147 (girls: *n* = 75, 51%; boys: *n* = 72, 49%) of them undergoing the cycling skills tests (OBS) and the training (Table 1). Some children were unable to take part in training due to injury; one child was unable to cycle at all. A total of 11 classes took part in the study. Of the 147 respondents who took part in the cycling test, 56.5% came from rural structures (400 inhabitants to 2500 inhabitants) and 43.5% from a small town (16,000 inhabitants). The survey with the children was administered in the classroom using a paper-and-pencil questionnaire (PAPI), with step-by-step instructions provided to ensure clarity and consistency. The children were told that both the survey and participation in the cycling test were voluntary, that it was not an examination, and that they could stop at any time if they wished. Informed consent was obtained from the parents of all participating children, and their willingness to engage in in-depth interviews was sought during parent–teacher meetings. Additionally, in advance of the study, consent was obtained from the Lower Austrian Education Authority.

In-depth interviews were conducted with 31 parents, some of which were held face-to-face while others were conducted via telephone. The parents were between 30 and 50 years old. There was only one father among the parents surveyed; all other participants were mothers. Additionally, eight teachers from the participating classes were interviewed, with a mix of face-to-face and telephone interviews conducted for this group as well.

### 2.2. Instruments

#### 2.2.1. Questionnaires

The children’s questionnaire (Questionnaire S1) contained questions mainly on their travel behavior, self-assessment of cycling skills, and attitudes. It was partly designed based on self-completion questionnaires, with which we have had positive experiences in interviewing children and youths [36]. We used pictograms (for example, for travel modes and attitudes) following mood icons from Westman and associates [37] and very simple wording. Questions on bicycle and scooter ownership, parents’ trip permissions, and family habits related to cycling were asked with simple yes/no questions. The children were supervised while filling in the questionnaire. Children stated the mode of transport used in the morning on the reporting date. To obtain correct measurements although the cycling test was on the same day, the children were instructed to take their bicycles to school the day before. However, at some schools, it was difficult to park the bicycles overnight. Therefore, it can be assumed that some children unusually used the bicycle on the reporting date or were car passengers, when parents brought the bicycle and the child by car. To consider this, children also reported the frequency of use on “normal” school trips ((nearly) always, frequently, sometimes, (nearly) never) for each given travel mode (car passenger, bus/train, scooter, bicycle, walking). Smileys and a 5-point scale were used for their self-assessment on cycling skills with two items: (i) “How good are you at cycling?” and (ii) “How comfortable/safe do you feel when you are cycling?”. Furthermore, their individual preferences for travel modes on school trips and leisure trips were surveyed. We also asked if the children like to be physically active (yes/no) and if they would like to get more exercise (yes/no). Other aspects of the questionnaire that are not reflected in the results are not described here.

The parental questionnaire (Questionnaire S2) included preset and open questions to examine the parental attitudes towards their own and their children’s bicycle use in daily mobility. The questionnaire can be structured in the following subject areas: (i) general characteristics of child and parent (such as driving license, availability of travel modes, sociodemographic characteristics); (ii) daily mobility patterns of parent and child; (iii) the child’s learning process how to ride a bicycle and the parental encouragement in promoting their child’s bicycle use; (iv) its actual cycling skills and habits; (v) the child’s physical activity level; and (vi) the parent’s attitudes regarding physical activity, active travel modes, and allowances. Also, the relationship between active travel and the well-being of their child was surveyed. The following description of the parental questionnaire is limited to the parts that are relevant for the following analysis.

Cycling skills: Parents’ perceptions of the children’s cycling skills were assessed with ratings based on a 5-point scale according to school grades with which parents are very familiar.

Physical activity (PA) level: Sports participation was evaluated by asking which sports the child regularly participates in during leisure time. Parents also reported the number of days and hours per week with moderate-to-vigorous physical activity (MVPA) (“On how many days is your child physically active so that the child is out of breath?”). The parents also expressed their opinion on adequate physical exercise (hours per day). They evaluated statements on the child’s bodily fitness and their PA level. They also assessed how their children feel when they use active travel modes a lot or seldom a day.

Parental encouragement: In order to learn more about the relationship between a child’s cycling skills and the influence of parents, we asked open questions about the child’s learning process of how to ride a bicycle. The parents described the child’s age, involved persons, and the way how the child was taught to ride a bicycle. We also asked if parents have been practicing cycling with their children on the road in preparation for the cycling test and if they plan to practice cycling on the road after the cycling exam (including reasons).

The interviews with teachers aimed at providing information on the (social) behavior and the performance of children in school. Normally, in Austrian primary schools, teachers know the children very well as they are together throughout the entire school day over four consecutive years. Because school performance is a particularly sensitive issue, the children were only evaluated as “rather above average” and “rather below average”.

#### 2.2.2. Cycling Test

We developed a cycling test to assess the cycling skills of the children in our sample. In contrast to the cycling exam, which also contains theoretical tasks, we focused on a physical test where children had to cycle through a course. The elements of our course are in conformity with a regulation of the Federal Ministry of Education, in which practical minimum requirements for passing the cycling exam are mentioned as well as unacceptable mistakes [38]. However, the specific test realization and the evaluation procedure are not defined in detail. During the voluntary cycling exam, cycling skills are not evaluated quantitatively, only the main mistakes are noted. The course in this study was developed based on existing studies in this field. The test stations (e.g., emergency and target breaking, slalom) followed examples from [39,40,41]. However, due to administrative reasons, it was necessary to keep the expenses as low as possible. Therefore, it was not possible to carry bulky equipment, for example, major obstacles or rental bikes. In this study, the cycling test consisted of seven stations to test for 10 selected basic cycling skills that children should manage to cycle safely in right-hand traffic (Table 2).

The key subjects addressed were (i) basic bicycle handling skills (such as braking, adjusting speed, balancing, and steering independently from direction and ground conditions) and (ii) physical safety skills to correctly maneuver through traffic (such as turning the head, signaling before turning, and braking to stop at traffic lights). Following other studies in this field [42,43,44], one special focus was on correctly turning left at intersections: In order to provide conditions as real as possible, children had to cycle on a public road and turn left to the school grounds or a parking lot. These conditions could be met at all but one school (where only school grounds had to be used). The main criteria for the assessment were the look back over the left shoulder, hand signaling, and driving position in the lane as well as the shape of the cycling curve when turning left.

The test was carried out on asphalt surfaces except for three test stations at one school (solid gravel ground). The children were instructed by the evaluators on how to perform at the test stations but were not allowed to practice. All of the stations were inspected carefully together. After that, the children performed the course one by one. The same two persons were present at all cycling tests and scored the children’s riding skills. The children were assessed alternately by the evaluators; a child’s cycling skills (before/after the training) were rated by the same person. Two evaluators were involved because the assessments required a high level of concentration; this also ensured an optimal flow between the consecutive runs of the children. In order to objectify the assessment as far as possible, the two evaluators assessed several children together in a pre-test. As mentioned above, there is no standardized assessment procedure that could be used. Therefore, a 5-point scale was used to assess the child’s general performance at each station, whereby 1 indicates the best and 5 the worst evaluation. The evaluation items and criteria were defined individually for each cycling skill (Appendix A, Table A1). The serious mistakes defined in the regulation of the cycling exam were considered. In the event of a fall, evaluation was set to 5 in any case. The use of a 5-point scale following the logic of Austrian school grades helped the evaluators to decide very quickly. The overall cycling skill score per child was calculated as a mean value over all cycling skills. According to this approach, we also calculated mean scores for left turn and braking. This means that we treated the allocated grades as interval-scaled, which is often performed when psychometric scales are used, for the purpose of simplification. This also allows us to compare these “objective” assessments to the ratings of parents and children, which also used 5-point scales (parents: school grades, children: smileys). For the objective overall cycling skill score, this also indicates that single poor scores can be compensated with good ones. For a general evaluation, this is adequate, as our test was not the basis to issue a cycling license (for which, for example, exclusion criteria in combination with a threshold of scores would be a better option).

#### 2.2.3. Cycle Training

Following the completion of the cycling test, a cycle training session was conducted. This training encompassed a series of exercises aimed at enhancing the children’s cycling skills, coupled with personalized feedback. The exercises comprised activities focused on improving balance, along with engaging in various cycling-related games. The entirety of the cycle training session lasted approximately 1 h. Due to constraints on space, a detailed description of the training activities is not provided here.

Subsequent to the training session, the children revisited the stations of the cycling test for another round of assessment.

## 3. Results

This section presents selected results of the study, highlighting key findings on children’s mode choice and preferences, their cycling skills (based on the assessment of evaluators, their self-assessment, and their parent’s assessment), and the relationship between cycling skills and children’s physical activity levels, and academic outcomes. The impact of bicycle training on children’s skills was also evaluated.

Regarding their mode choice for school trips, 14% of the children reported that they (almost) always (3%) or often (11%) use a bicycle on a normal school day (25% sometimes, 61% never) (Table 3). On the day of the survey, a higher proportion used the bicycle because they were asked to do so. When asked about their attitudes regarding different travel modes (on a 5-point scale), children rated active forms of mobility very highly: Over 90% of children said that cycling was a “very cool” or “good” way to travel (scooter 70%, walking 65%). The car and public transport cannot keep up with the positive overall picture of the other active forms of mobility: 22% of children rated the car and more than 38% the bus and train as “okay” or “bad”. Children’s positive attitudes towards active travel modes are also reflected in their preferences for using them for school and leisure trips. Almost 60% of the children would prefer to travel by bicycle (Table 3).

Table 4 shows the mean scores of children’s cycling skills (before cycle training), which are on a (very) good level between 1.1 and 2.5 (ranked on a 5-point scale). The values indicate that the test stations “left turn” and “slalom” caused difficulties compared to the other tasks. In contrast, the children mastered cycling on difficult terrain and in a straight line quite easily. Within the category “braking”, emergency braking received the worst score, but 1.9 is still on a good level. There were no significant differences in cycling skills between girls and boys and between rural and urban schools.

As outlined above, the children who took part in the cycling task were asked about their self-assessment of their cycling skills before they passed the cycling course. In terms of bicycle handling, almost all children (98%) rated their cycling skills as “very good” or “good” (on a 5-point smiley scale) (Table 5). Similar results were found for children’s subjective feeling of safety while cycling. For both assessments, there were no noticeable differences between girls and boys.

**Figure 1:**
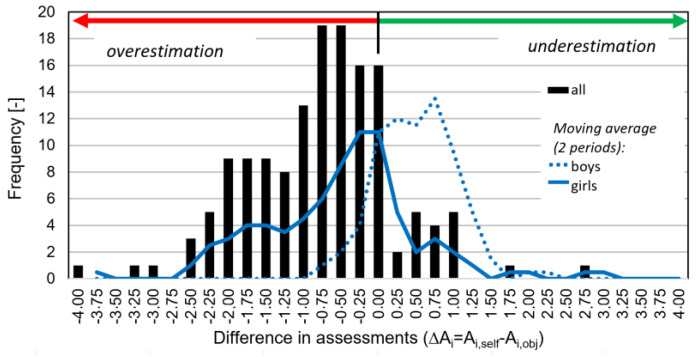
Frequencies of differences in children’s self-assessments of cycling skills (5-point scale from 1 = high skills to 5 = low skills). A_i,self_ = self-assessment of child i, A_i,obj_ = objective assessment of child i while passing the cycling course (*n*_all_ = 147).

However, equating the two evaluation scales shows a clear contradiction between the objective and subjective evaluation of cycling skills. Children overestimated their cycling skills (in 77% of cases); 40% overestimated their skills by even one grade or more (Figure 1). Interestingly, girls overestimated their cycling skills much more than boys who rather underestimated (mean of deviations from the objective assessment: ∆A_girls_ = −0.6, ∆A_boys_ = +0.4).

**Figure 2:**
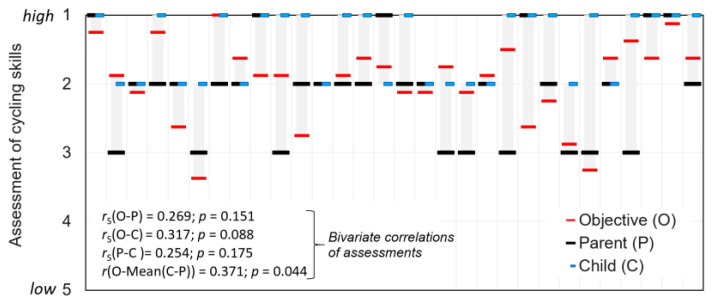
Comparison of children’s cycling skills as observed in the cycle course (O), compared with assessments from children (C) and parents (P), *n* = 30.

In addition to the objective and the children’s subjective assessment of their cycling skills, the parents’ perspectives could also be included, based on the interviews. However, it should be noted that the sample size is small as the information is only available for a sub-sample of 31 children. Figure 2 shows a comparison of the children’s cycling skills as observed during the cycling parcourse (O) with the children’s (C) and parents’ (P) statements. The results indicate that most children and parents over- or underestimate the children’s cycling skills, sometimes quite significantly. The results show low bivariate correlations between the three ratings, ranging from 0.25 to 0.32. The highest correlation (*r* = 0.371) exists between an objective and a mean assessment of C and P.

As mentioned above, we also collected child-related information, such as the physical activity of the child, during the interviews with the parents. According to their parents, children in the sub-sample engage in moderate or vigorous physical activity for 7.9 h per week, or an average of 1.1 h per day. At first sight, this would mean that, surprisingly, the children meet the weekly PA recommendations of the WHO. However, if we take a closer look, the differences between the children are very high (range between 2 and 21 h per week) and only 43% meet the recommendations. When the “number of active days per week” reported by parents is taken into account, 65% of the children do not meet the recommendations, as PA is accumulated mainly at weekends.

**Figure 3:**
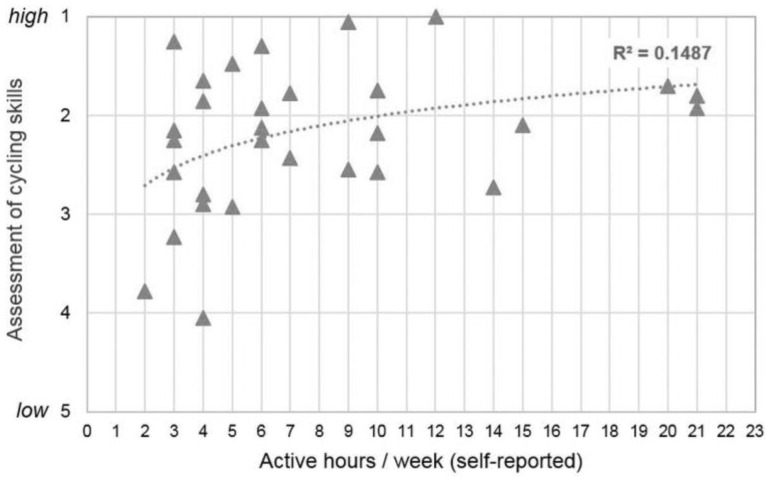
Scatter diagram of physical activity (hours per week), stated by parents (*n* = 30) and overall cycling skills of primary school children, logarithmic trend line with coefficient of determination.

The detailed analysis shows that there is a positive relation between PA and cycling skills of the children: Negative bivariate correlations throughout each skill (*r* = −0.07 for “left turn” to −0.42 for “braking”) indicate that the higher the reported number of active hours per week, the better the overall cycling skills (*r*_overall_ = −0.31) (Appendix A, Table A2). As mentioned above, cycling skills were rated following the logic that lower values imply better skills. Figure 3 gives a scatter diagram and a logarithmic trend line on the relation between PA measured with active hours per week and the overall objective assessment of cycling skills. A logarithmic dependency seems to be plausible as above a special threshold, no improvement can be achieved anymore. The results indicate relations in the expected direction but on a very low level. The number of kilometers cycled in the last seven days correlated at a lower level with children’s cycling skills. The mostly negative correlation coefficients indicate that the higher the number of kilometers cycled, the better the objective cycling skill score (Appendix A, Table A2).

We divided the children into two groups according to whether they met the WHO recommendations or not and compared their objective cycling skills (mean score). Children who met the recommendations had better cycling skills (We used the weighted mean scores of cycling skills for which problematic stations (turning left, breaking, and slalom) were weighted higher.) (*M* = 1.94) than the other group who did not meet the recommendations (*M* = 2.40). However, the differences are not significant (t(28) = 0.1.742, *p* = 0.093; *r* = 0.35, *n* = 30). The small sample size should be noted.

A comparison of the children’s observed cycling skills at the end of the course before and after the cycle training showed a highly significant improvement in mean performance scores from *M_before_* = 1.77 to *M_after_* =1.36 (paired *t*-test, *t* = −6.992, *p* < 0.001, *n* = 147) (Appendix A, Table A3). The effect size according to Cohen [45] is *r* = 0.91 and thus corresponds to a strong effect. The detailed scores after the training show that “turning left” is comparatively still the most complicated task.

We also analyzed the relationship between cycling skills and teachers’ evaluations. In terms of school performance, teachers categorized 23% of the children as performing “below average”, 72% as “above average”, and 5% as having no assessment. Bivariate correlations show the tendency that the cycling skills are higher (low score) the higher their school performance is estimated (Table 6). Furthermore, children who stated that they “would like to be more physically active” (which refers to 69%) had worse school performances and were less concentrated during school lessons.

No correlation can be found between school performance and sufficient exercise. It should be noted here that information on (inadequate) physical activity is only available for a very small number of children and is based on parents’ judgments, which—in terms of cycling skills—do not correlate well with objectively rated cycling skills.

Based on their impressions, parents stated that their child has better well-being on days when they ride a bicycle or a scooter a lot. It seems to be “more confident”, “balanced”, and “happy”. In contrast, parents observe that their child is “restless”, “annoyed”, and “bad-tempered” on days when they travel as car passengers. This was confirmed in statements parents gave during the in-depth interviews, for example:

*“Although he travels actively to school for the most time, his urge to move is very high at school. But he is happier when he got home from school walking than if he is brought home by car.”*

*“[The child] is more confident, less stressed (…)”. “(…) no behavioral problems [Note: if daughter is mainly actively out and about], but she is unbalanced, fidgety, in a bad mood when we mainly used the car.”*

*“He is less concentrated, easily irritable—he is also daydreaming and not receptive on these day [Note: with low use of walking and/or cycling].”*

*“He feels good and is mentally fit. (…) Only driving as a car passenger is very burdensomely for him. Then a balance is needed, for example a bicycle tour after a long car drive to a holiday resort.”*

In addition to these behavioral impacts, 94% of the interviewed parents (fully) agree that physical activity has a positive impact on school performance.

## 4. Discussion

The findings of this study offer several important insights, particularly regarding primary school children’s perceptions of their cycling skills and the relation between physical activity (PA) levels, cycling abilities, and academic performance. Utilizing a mixed-methods approach, we combined direct observations of children’s cycling skills with self-assessments from children and parents, revealing significant biases in skill overestimation. While we observed a weak association between PA levels and cycling skills, supporting the importance of meeting WHO recommendations, the results lacked statistical power. In addition, the study links cycling skills with broader behavioral and academic outcomes, providing valuable insights for educators, policymakers, and researchers. Overall, our results show children’s positive attitudes regarding cycling. Despite low rates of cycling to school, cycling dominated as children’s preferred mode of transport to school and leisure destinations. This aligns with findings from other studies [46,47] and emphasizes the importance of encouraging children to develop their cycling skills so that they can fulfill their aspirations.

Our observations revealed that especially “left turn” and “slalom” seem to cause some difficulties for the children, although the overall objective assessments were generally on a good level ranging from 1.09–2.54 on a 1-to-5 scale. These findings appear to be consistent with results from other studies, e.g., [42], that found that turning maneuvers (and also braking processes) pose difficulties for children. As mentioned above, turning left is one of the most complex processes of cycling in daily life, demanding good psychomotor coordination. In terms of braking, the task “emergency braking” in our study was also one of the lower scores. Cycling on difficult ground conditions seemed to be an easier task for the children in this study, which is in line with findings for 4th graders from Belgium who showed the best performance in cycling over obstacles—the lowest score was found for signaling left and right while cycling in a straight line [48]. The overall good scores achieved by the children on the course could be due to the fact that the participants live in rural areas or in small towns. Cycling skills are likely to be significantly better here than in the city [32,33]. In the city of Vienna, fewer and fewer pupils are taking the voluntary cycling test and failure rates are high (2024: 45%) [49]. Another explanation could be that parents consciously practiced cycling with their children in preparation for the workshop. During the interviews, it was noted that 45% of the children had cycled on 1–2 days in the past week, 23% on 3–4 days, and 13% on 5–7 days. This contrasts with the information regarding the children’s general travel habits and may suggest that additional practice was undertaken specifically for the workshop. Correlations between the kilometers cycled in the last few days and cycling skills indicate that practice has a positive effect.

It is challenging to classify our sample in terms of meeting the WHO physical activity recommendations, as no representative data are available for Austria for 3rd- and 4th-grade primary school students. In our sample, 35% meet the recommendations, which is roughly comparable to the proportion of 11-year-olds (31%) [50]. This information for our sample is based on parents’ assessments. It should be noted that face-to-face interviews may introduce bias, as parents might be inclined to provide socially desirable responses. We assume that the small sample of volunteering parents predominantly consisted of those who are generally concerned about their children’s well-being and more actively involved in promoting physical activity. Consequently, it is likely that only parents with a strong affinity for active lifestyles volunteered to participate in the in-depth interviews, resulting in a potential self-selection bias. An indication for this is that 64% of the parents stated to use their bicycle (nearly) every day or 2–3 times a week. This is also suggested by the information provided by the parents at which age their children learned to ride a bicycle: We found that half of the children learned to cycle at the age of 3 years, one-third at 4, and 16% at 5 years; one child could already cycle with 2 years. All parents interviewed seemed to be really concerned with teaching their children to ride a bicycle in the early years. None stated that their children learned cycling late. Furthermore, 87% of the parents reported to exercise cycling in preparation for the official bicycle test. They described to cycle selected routes together for training. However, we have no more detailed information on the framework of these trainings. It should be noted that there is a bias in the sample of parents, as almost exclusively mothers took part in the interview. However, it is also possible that mothers are in a better position to assess their children, as they are usually responsible for childcare to a much greater extent than fathers. The schools that volunteered for workshops on cycling may reflect a general interest in the topic but could also suggest a self-selection bias.

Notably, our research revealed a tendency for many children (over 40% of the children), especially girls, to (grossly) overestimate their bicycling abilities, which became evident during the assessment of their skills under real riding conditions. Furthermore, the parents’ assessments clearly differ from the objective observations. There is no comparable literature to categorize these results. On the day of the surveys, we assume that the children could have been very excited on this special “biking day”, expressing this excitement in an overestimation of their self-reported regular bicycling activities and abilities.

Nevertheless, it should be noted that this significant overestimation of one’s abilities and misperception of safety, particularly in real-world traffic situations, implies some risks, especially, if parents also misjudge the children’s capabilities and allow them to use their bicycles unsupervised on the road. Even more, this clearly underlines that (i) the children have difficulties assessing their capabilities realistically and (ii) a high need for training under the supervision of parents.

Although existing literature suggests that physical activity enhances children’s psychomotor skills [51] and that bicycling skills are associated with motor competencies [52], our study did not yield statistically significant results. Children with higher levels of physical activity only showed a tendency toward better cycling skills. Furthermore, as previously mentioned, the association with cycled kilometers might reflect a short-term training effect. This outcome may be attributed to the limited sample size, influenced by parental disinterest in participating in in-depth interviews. Parental misjudgments may also have influenced the results.

Similarly to Dadaczynski [53] who analyzed the impact of health on education, our sample showed an association between higher cycling skills and better teacher ratings. However, no statistically significant results were found. This may be because teachers provided only rough estimates of school performance and behavior, with the majority of children being rated as above average.

A main finding of our study is that (even a short) cycling training led to significantly better cycling scores. This finding is in line with the findings of Ducheyne and associates [54] who found a significant positive effect of cycling training courses on children’s cycling skills. If even short training can improve performance so much, it underscores the importance of incorporating regular, structured cycling practice into children’s routines. However, it is crucial to recognize that the benefits of such training may be short-lived without consistent practice. Extending cycling training beyond primary school could further enhance long-term skill development. However, it could be expected that encouraging children to lead an active lifestyle, with a significant increase in PA over 1 h per day, would result in significantly better cycling skills and better orientation in real traffic conditions.

Besides the small sample size, this study is limited by the lack of data on the reliability and validity of the measures used, which should be addressed in future research. It is imperative to implement standardized psychometric tests within the experimental design to assess children’s overall motor development accurately. Additionally, recording and evaluating children’s physical activity through activity diaries can offer unbiased insights into their actual activity levels. Methodologically, conducting more interviews with parents (including fathers) and enhancing the evaluation of children’s school performance by teachers can further refine the data and results. Furthermore, embarking on projects involving extensive cycle training for children, ideally accompanied by coaching processes to facilitate unaccompanied cycling in real traffic conditions, could provide valuable insights into discrepancies observed between self-reported physical activity levels and actual bicycling performance, particularly concerning maneuvers such as left turns.

The transferability of our results to other countries or regions is questionable, because cultural, infrastructural, and societal factors influence the extent of parents’ perceptions of safety [55,56,57] as well as cycling adoption and skill development. In cycling-friendly regions like Northern Europe, positive attitudes and strong skills reflect supportive infrastructure, while car-centric cultures may face barriers to active travel. Discrepancies between subjective and objective skill assessments may also vary with cultural perceptions of safety and independence.

Future researchers investigating children’s cycling skills and their relationship with physical activity should consider adopting a more robust methodological approach. This includes using standardized psychometric tools to assess motor development, employing objective measures such as activity trackers to record physical activity levels, and conducting a higher number of parent and teacher interviews to gain deeper insights into children’s behavior and performance. Larger sample sizes are crucial to enhance the generalizability of findings, and longitudinal studies could help explore the long-term effects of cycling training on motor skills, safety in traffic, and overall development. Additionally, integrating real-world cycling tests under supervised traffic conditions could provide a more accurate assessment of children’s abilities and the effectiveness of training interventions.

## 5. Conclusions

This study highlights significant overestimation biases in both children’s and parents’ assessments of children’s bicycling skills. These overestimations reveal a critical gap in self-awareness and real-world cycling competence, emphasizing the need for supervised, structured cycling training. Parents should be actively involved in these training programs to ensure both parties develop a realistic understanding of the child’s abilities and limitations, particularly in traffic situations. Specifically, challenges like left turns and emergency braking, which were found to be particularly difficult for children, underscore the importance of targeted skill development under safe, controlled conditions.

Our findings suggest that higher physical activity levels may be associated with better cycling competence and slight academic benefits, although these relationships were not statistically significant. While most children in our sample did not meet the WHO’s physical activity recommendations, it is clear that physical activity alone does not fully capture the complexities of cycling proficiency, and dedicated cycling training is essential.

Given the concerning trend of decreasing physical activity levels among children, interventions aimed at promoting both cycling and overall physical activity are crucial. It is particularly important to focus on creating safer cycling environments for school commutes, as inadequate traffic conditions remain a significant barrier to children’s participation in cycling, despite many children expressing a preference for cycling as a mode of transport. Addressing these concerns can help instill confidence among parents and encourage children to adopt cycling as part of their daily routines.

Future research should prioritize standardized motor skill assessments, objective physical activity measures, and larger sample sizes to validate findings. Longitudinal studies and real-world cycling tests are recommended to assess the long-term effects of cycling training on children’s skills, safety, and overall development. Furthermore, initiatives aimed at improving road safety education, enhancing cycling infrastructure, and implementing widespread cycling programs are essential for empowering children to become safe, confident road users. These efforts can also contribute to fostering a broader culture of active mobility and healthier lifestyles.

## Figures and Tables

**Table 1:** Data sources and method of data collection (PAPI = paper-and-pencil interview; OBS = observations; IDI = individual (in-depth) interview; F2F = face-to-face interview; TI = telephone interview).

School	Children (PAPI)	Cycling Skill Tests (OBS)	Parents (IDI: F2F + TI)	Teachers (IDI: F2F + TI)
A	25	24	3	1
B	12	10	1	0
C	23	23	5	1
D	27	26	8	2
E	65	64	14	4
Sum	152	147	31	8

**Table 2:** Cycling test stations.

Station	Description	Schematic Sketch
1	Left turn: Children had to cycle on a public road and turn left to the school ground or a parking place. Children need to look over their left shoulder, make the correct hand signal, swerve towards the middle of the lane, and should not cut the curve.	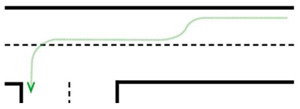
2	Cycling in a narrow lane: This task is to cycle between two parallel lines without pedaling, strong steering motions and hitting the cones. The straight path of 6.00 m in length and 0.35 m in width is marked with 9 cones at each line.	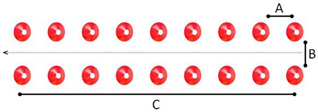 A = 0.50 m; B = 0.35 m; C = 6.00 m
3	Targeted breaking: Children cycle on a 20.00 m long acceleration lane and need to come to a controlled stop at the marking line (stopping sign).	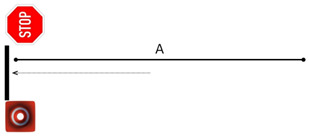 A = 20.00 m
4	Cycling slalom: 13 cones are placed on a curved track with varying gaps. The first 2 gaps and the gaps between cones 7, 8, and 9 are 2.50 m wide; the other gaps are 1.80 m wide. Children cycle in and out of cones, staying close to them without touching them. Children should take all the slaloms and brake sufficiently to reduce speed.	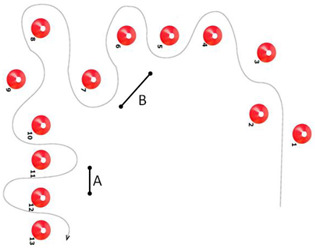 A = 1.80 m; B = 2.50 m
5	Emergency braking: Children cycle along a 20.00 m long acceleration lane and have to stop immediately after an acoustic signal. A fast reaction and an even-breaking process are required. Children need to come to a controlled stop.	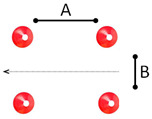 A = 20.00 m; B = 2.00 m
6	Cycling over obstacles: Children cycle over four obstacles that are placed in a straight line behind each other. The wooden bars are 1.20 m long with rising width along the track. The first bar is 2 cm high, and the further ones are 2.50, 3.00, and 4.00 cm. The straight track of 7.00 m in length is marked with cones. The children need to maintain their balance and speed while cycling over the obstacles. This task is to simulate kerbstones.	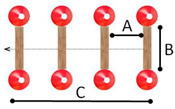 A = 1.50 m; B = 1.20 m; C = 7.00 m
7	Cycling on uneven surfaces: Children cycle over pieces of wood (5 cm diameter) and rope (about 1 cm diameter). While cycling over the obstacles, the children need to maintain their balance and speed. The track is 5.00 m long and 2.00 m wide and demarcated with cones. This task is to simulate dirty and gravel road sections.	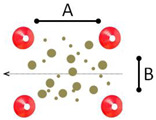 A = 1.50 m; B = 1.20 m

**Table 3:** Children’s mode choice (i) on the reporting date, (ii) on a typical school day, (iii) as the preferred mode for their school trips, and (iv) as the preferred mode for their leisure trips (*n* = 147).

Travel Mode	(i) On the Reporting Date	(ii) On a Typical School Day	(iii) Preference School Trip	(iv) Preference Leisure Trips
Walking	19%	31%	12%	14%
Scooter	5%	12%	12%	18%
Bicycle	34%	3%	60%	59%
Public transport	18%	23%	9%	1%
Car passenger	24%	24%	7%	7%

Note: For (ii), we used children’s answers “(nearly) always”, as this question was asked on a 4-point scale (nearly) always, often, sometimes, (nearly) never.

**Table 4:** Children’s “objective” cycling skills for different tasks on a 5-point scale (1 = best, 5 = worst; mean values of assessment and standard deviation).

Task	Cycling Skills (Test Station Number)	Mean (SD)
Left turn	Looking over left shoulder (1)	1.90 (1.310)
	Hand sign (1)	1.99 (1.285)
	Swerving to the middle of the lane (1)	2.15 (1.235)
	Curve (1)	2.24 (1.235)
Straight-ahead path	Cycling in a narrow lane (2)	1.24 (0.764)
Braking	Velocity (3)	1.71 (1.105)
	Stopping accuracy (3)	1.68 (1.194)
	Emergency braking (5)	1.90 (1.109)
Slalom	Cycling slalom (4)	2.54 (1.386)
Ground conditions	Cycling over obstacles (6)	1.10 (0.346)
	Cycling on uneven surfaces (7)	1.09 (0.421)

**Table 5:** Children’s “subjective” cycling skills on a 5-point scale (1 = best, 5 = worst; mean values of assessment and standard deviation), *n* = 147.

Category	Sex	Mean (SD)
Overall assessment of cycling skills	Girls (*n* = 75)	1.32 (0.640)
	Boys (*n* = 72)	1.31 (0.493)
Feeling of safety while cycling	Girls (*n* = 75)	1.36 (0.729)
	Boys (*n* = 72)	1.33 (0.581)

**Table 6:** Bivariate correlations between (i) cycling skills (mean score, the lower the higher the cycling skills) and (ii) children’s wish to be more physically active (0 = no, 1 = yes) and teachers’ evaluations of children (academic performance: 0 = below average, 1 = above average; others: 0 = does not apply, 1 = applies), Pearson correlations, *n* = 140–145.

	(i) Cycling Skills		(ii) Wish “More Active”		
Teachers’ Evaluations	r	Sig. (2-Tailed)	r	Sig. (2-Tailed)	N
School performance	−0.210	0.013 *	−0.197	0.020 *	140
Happy/confident	−0.029	0.732	−0.006	0.942	141
Concentrated/mentally present	−0.138	0.098	−0.243	0.003 **	145
Restless, easily irritable	−0.020	0.814	0.092	0.274	142
Integrates into the group	−0.105	0.210	−0.030	0.719	144

Note: * *p* < 0.05; ** *p* < 0.01.

## Data Availability

The raw data supporting the conclusions of this article will be made available by the authors upon request.

## Supplementary Materials

The following supporting information can be downloaded at https://www.mdpi.com/article/10.3390/children11121556/s1, Questionnaire S1: Children questionnaire. Questionnaire S2: Parent questionnaire. Please note that these are translated versions; the original versions used in the study are in German.

## Appendix A

**Table A1:** Cycling skills and criteria for scoring on a 5-point scale.

Topic	Cycling Skills	Evaluation Items and Criteria for Scoring ^1^
Make a turn to the left	Looking over left shoulder (station 1)	(1) Looking back correctly (2) Looking back only indicated (3) Looking back with a strong steering motion (4) Looking back with changes in direction (5) Without looking back
	Hand sign (station 1)	(1) Correct hand sign (2) Hand sign too short (3) Hand sign vaguely (4) Hand sign with strong steering motion (5) No hand sign
	Swerving towards the middle of the lane (station 1)	(1) Position in lane correctly (centered, in time) (2) Position in lane centered, but too late (3) Position too far to the right (4) Position too far to the left (5) Turning left without changing position in the lane
	Curve (station 1)	(1) Curve not cut, right position (2) Curve not cut, position too far to the right (3) Curve not cut, position too far to the left (4) Curve cut slightly (5) Curve cut significantly
Straight-ahead path	Cycling in a narrow lane (station 2)	(1) Passing through correctly (2) Passing through with pedaling (3) Hitting a cone (4) Knocking down a cone (5) Leaving the track
Braking	Target braking (station 3)	Velocity: (1) Adapted speed (2) Speed too slow (3) Speed too fast (4) Speed much too slow (5) Speed much too fast Stopping accuracy: (1) Correct position (2) Position too far back (3) Position much too far back (4) Position too far forward (5) Position much too far forward
	Emergency braking (station 5)	(1) Breaking process correctly (2) Breaking process too hesitant (3) Breaking process too abrupt (4) Breaking process much too hesitant (5) Breaking process much too abrupt
Curved path	Cycling slalom (station 4)	(1) Passing through correctly (2) Passing through with a strong steering motion (3) Passing through just about acceptable (4) One foot on the ground (5) Obstacles omitted, Leaving the track
	Cycling on an uneven surface (stations 6 and 7)	(1) Passing through correctly (2) Passing through with a strong steering motion (3) Passing through just about acceptable (4) One foot on the ground (5) Obstacles omitted, Leaving the track

^1^ Note: In the event of a fall, evaluation was set to 5.

**Table A2:** Bivariate correlations between cycling skill score per task (a lower score means higher skills) and (i) children’s active hours/week and (ii) km cycled during the last seven days, *n* = 30.

Task	Active Hours per Week	Cycling km Last 7 Days
Looking over left shoulder (station 1)	0.145	−0.186
Hand sign (station 1)	−0.132	0.044
Swerving towards the middle of the lane (station 1)	−0.108	−0.227
Curve (station 1)	−0.112	−0.200
Cycling in a narrow lane (station 2)	−0.136	−0.148
Target braking (station 3)	−0.252	−0.014
Emergency braking (station 5)	−0.404	−0.289
Cycling slalom (station 4)	−0.341	−0.025
Cycling on an uneven surface (station 6)	−0.133	−0.026
Cycling on an uneven surface (station 7)	−0.133	−0.026

**Table A3:** Change of cycling skills before and after the cycling training, paired samples statistics.

	M	SD	M_a-b_	SD_a-b_	t	Sig. (2-Tailed)
After (a)	1.3636	0.3355	−0.4000	0.1897	−6.992	0.000
Before (b)	1.7636	0.4632				
