# Supplementary material for: On the Road to Safety: Examining Children’s Cycling Skills and Physical Activity Levels
Source: Children (Basel). 2024 Dec 22;11(12):1556. doi: 10.3390/children11121556 (PMC11727309; doi:10.3390/children11121556)
Supplement: Supplementary file 1 [file children-11-01556-s001.zip › children-3371529-supplementary.pdf]

Start number:__________

date/school:

____

By the way: This is not an exam - you can't do anything wrong! Nobody who knows you will look at your questionnaire afterwards. If you do NOT want to fill in something, skip the question!

I am a: Boy ☐ Girl ☐ Other / I do not want to say ☐

---

1. What mode of transport did you use to get to school today?

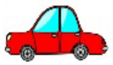
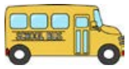
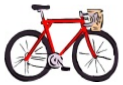
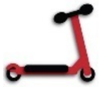
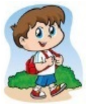

Car Bus/train Bicycle Scooter On Foot

2. Which mode of transport would you like to use to get to school if you could choose it yourself?

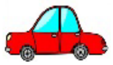
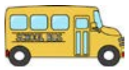
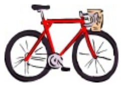
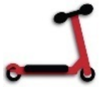
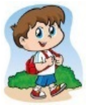

Car Bus/train Bicycle Scooter On Foot

3. Which mode of transport do you prefer to use in your free time?

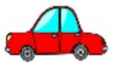
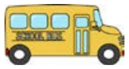
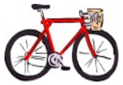
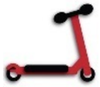
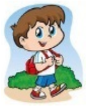

4. Car Bus/train Bicycle Scooter On Foot

Note: The original version of this questionnaire is in German. This is a translated version.

5. How often do you use this mode of transport on your way to school?

	(nearly) always	often	sometimes	(nearly) never
**Car** 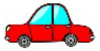	☐	☐	☐	☐
**Bus/train** 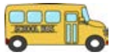	☐	☐	☐	☐
**Bicycle** 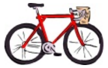	☐	☐	☐	☐
**Scooter** 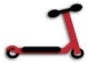	☐	☐	☐	☐
**Walking** 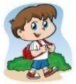	☐	☐	☐	☐

Note: The original version of this questionnaire is in German. This is a translated version.

6. What do you think of this mode of transport?

	Really cool 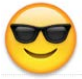	*good* 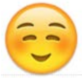	*average* 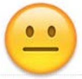	*Bad* 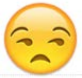	*Really bad* 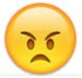
**Car** 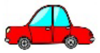	☐	☐	☐	☐	☐
**Bus/train** 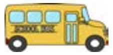	☐	☐	☐	☐	☐
**Bicycle** 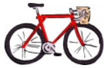	☐	☐	☐	☐	☐
**Scooter** 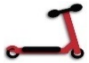	☐	☐	☐	☐	☐
**Walking** 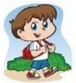	☐	☐	☐	☐	☐

Note: The original version of this questionnaire is in German. This is a translated version.

7. Please answer the following questions!

	Yes ✔	*No* ✖
I have my own functioning bicycle.	☐	☐
I have my own functioning scooter.	☐	☐
I am allowed to be out on the road alone.	☐	☐
I go hiking with my family.	☐	☐
I cycle with my family.	☐	☐
I like to move/exercise.	☐	☐
I would like to move/exercise more.	☐	☐

8. Cycling

	Very good	*Rather good*	*average*	*Rather bad*	*Very bad*
How good are you at cycling?	☐	☐	☐	☐	☐
How safe do you feel when cycling?	☐	☐	☐	☐	☐

Note: The original version of this questionnaire is in German. This is a translated version.

9. How do you feel in the first lesson when you ...

	😄	☺️	😐	😒	😩
... **walked** to school?	☐	☐	☐	☐	☐
... **cycled** to school?	☐	☐	☐	☐	☐
... someone took you to school by **car**?	☐	☐	☐	☐	☐
... you travelled to school by **bus** or **train**?	☐	☐	☐	☐	☐
... you travelled to school by **scooter**?	☐	☐	☐	☐	☐

Note: The original version of this questionnaire is in German. This is a translated version.

10. How do you feel in the last lesson at school when...

	😄	☺️	😐	😒	😩
... **walked** to school?	☐	☐	☐	☐	☐
... **cycled** to school?	☐	☐	☐	☐	☐
... someone took you to school by **car**?	☐	☐	☐	☐	☐
... you travelled to school by **bus** or **train**?	☐	☐	☐	☐	☐
... you travelled to school by **scooter**?	☐	☐	☐	☐	☐

Thank you!

Note: The original version of this questionnaire is in German. This is a translated version.

Interview guide - In-depth interview

Date, place:____

Primary school (name, street, postal code, town): ________________

Duration [min]: ____

Persons present: ____

Interviewer: ____

Place of residence (street, zip code, town): ______________________

Introduction

Hello and thank you very much for agreeing to take part in a personal interview. The interview will take about 30 minutes. In particular, we are investigating topics such as choice of means of transport, well-being and cycling skills in order to derive measures for increasing children's road safety.

The research will be scientifically monitored by the Institute for Transport Studies at the University of Natural Resources and Life Sciences (BOKU), Vienna

The evaluation of the interview will be anonymised and your data will not be passed on to third parties. As it is very helpful for the evaluation, I would record the interview with a dictation machine with your consent. The recording will be deleted immediately after anonymized entry into the database.

Switch on the dictation machine

BLOCK A

	Adult	Child	Comments
**Your name**	☐	☐	
**Year of birth**	☐	☐	
Gender	*male* ☐<br>*female* ☐	*male* ☐<br>*female* ☐	
**Highest educational qualification**<br>No qualification (yet)<br>Secondary school without apprenticeship<br>Secondary school with apprenticeship, technical college<br>general qualification for university entrance<br>College, university, technical college	☐<br>☐<br>☐<br>☐<br>☐		*Please note:*<br>*formatting of columns one and two had to be changed due to translation.*
**Availability of properly functioning bicycle(s)**<br>➔ Numbers per person	☐	☐	
**Car availability**<br>➔ Number per household	☐		
**Driving license possession**	Yes . ☐ No .... ☐		
**Cycling license**<br>*(for children)*		completed .... ☐<br>passed .... ☐	*Required to ride a bicycle independently from 10 (9) years instead of 12 years*
**Season ticket/discount card for public transport**<br>Weekly, monthly or annual ticket<br>Advantage or discount card<br>*(multiple answers possible)*<br>none of the above	☐<br>☐<br><br>☐	☐<br>☐<br>☐ (text box)<br>☐	*Please note:*<br>*formatting of columns one and two had to be changed due to translation.*
**Distance** [km] Place of residence - elementary school	☐		
Place of residence - nearest public transport stop	☐		
⇨ Type of public transport at the next stop	☐		

Block B

**At what age did your child learn to ride a bike?**	
**How did your child learn to ride a bike?**<br>**(balance bike, bike with support wheels, scooter, ...)**	
**Who helped your child learn to ride a bike?**<br>**(Mom, dad, siblings, grandma, grandpa, ...)**	
**On how many of the last 7 days did your child cycle?**	
**How many kilometers in total?**	
**What were the purposes of your child's journeys?**	
**Would you let your child ride alone in traffic after passing the cycling test?**	**Yes.....☐** **No.....☐**<br><br>**What are the reasons for your answer?**
If no: ***Why not?***<br>*(multiple answers possible)*	**Too dangerous for my child** .... ☐<br>**There is no suitable infrastructure** .... ☐<br>**Risk of damage/theft is too high** .... ☐<br>**My child's cycling skills are not good enough** .... ☐<br>**Traffic rules are not yet sufficiently enforced** .... ☐<br>**Other reasons, namely** .... ☐

**Did you practice cycling in traffic with your child before the cycling test**	**No ...** ☐<br>**Yes ....** ☐<br>*If Yes:* **What was practiced and how often?**<br>**Where and under what conditions did these exercises take place?** (situation, location)
**Will you practice cycling in traffic with your child after the cycling test?**	**Yes ....** ☐<br>**No ...** ☐<br>*If Yes:* **What exactly? Where and under what conditions do you want to practice?** (situation, location)<br>*If Yes:* **Why not?**
**How high do you rate your child's cycling skills before the cycling training?** *(evaluation with school grades)*	**Very good** ☐ **Good** ☐ **Satifactory** ☐ **Sufficient** ☐ **Not sufficient** ☐ Don't know ☐
**Have you been cycling with your child after the bike training?**	**Yes ....** ☐ **No .....** ☐
**How high do you rate your child's cycling skills after completing cycle training?** *(Evaluation with school grades)*	**Very good** ☐ **Good** ☐ **Satifactory** ☐ **Sufficient** ☐ **Not sufficient** ☐

Block C

How often do you use the following means of transportation?

Adult	*(almost) daily*	*2-3 times a week*	*2-3 times a month*	*Less than once a month*	*(almost) never*
Walking	☐	☐	☐	☐	☐
Bicycle	☐	☐	☐	☐	☐
Bus / train	☐	☐	☐	☐	☐
Car driver	☐	☐	☐	☐	☐
Car passenger	☐	☐	☐	☐	☐
Moped / motorcycle	☐	☐	☐	☐	☐
scooter	☐	☐	☐	☐	☐

Child	*(almost) daily*	*2-3 times a week*	*2-3 times a month*	*Less than once a month*	*(almost) never*
Walking	☐	☐	☐	☐	☐
Bicycle	☐	☐	☐	☐	☐
Bus / train	☐	☐	☐	☐	☐
Car driver					
Car passenger	☐	☐	☐	☐	☐
Moped / motorcycle					
scooter	☐	☐	☐	☐	☐

Block D

**What sporting (physically active) activities does your child do regularly?**	
**On how many days per week is your child physically active so that they get out of breath?**	
**Approximately how many hours per week?**	
**How important is physical activity for your child in everyday life or during leisure time?**	**Very important ☐ Rather important ☐ Less important ☐ Not important ☐** Don't know ☐
**How important is it to you that your child walks or cycles as often as possible?**	**Very important ☐ Rather important ☐ Less important ☐ Not important ☐** Don't know ☐
**My child gets enough physical exercise if it can romp around for ________ hours/per day.**	
**How do you recognize that your child is physically at capacity?**	
**To what extent do you agree with the following statements?**	
**My child is physically fit.**	**Strongly agree ☐ Agree somewhat ☐ Disagree somewhat ☐ Disagree☐**
**Physical education is sufficient for my child's well-being.**	**Strongly agree ☐ Agree somewhat ☐ Disagree somewhat ☐ Disagree☐**
**Sporty or physically active children have better social skills.**	**Strongly agree ☐ Agree somewhat ☐ Disagree somewhat ☐ Disagree☐**
**Physical exercise has a positive influence on academic performance and mental fitness.**	**Strongly agree ☐ Agree somewhat ☐ Disagree somewhat ☐ Disagree☐**
**To what extent does it affect your child's well-being / behavior if he / she walks / rides a bike / scooter a lot in one day?**	
**To what extent does it affect your child's well-being / behavior if he / she is a passenger in a car a lot on a given day?**	

To what extent do you agree with the following statements?

If my child was mainly out and about with ... on one day, does he / she feel ...?	On foot	Bicycle / scooter	Bus / train	Car
healthy	fully agree☐☐☐☐fully reject	fully agree☐☐☐☐fully reject	fully agree☐☐☐☐fully reject	fully agree☐☐☐☐fully reject
Strong / energetic	fully agree☐☐☐☐fully reject	fully agree☐☐☐☐fully reject	fully agree☐☐☐☐fully reject	fully agree☐☐☐☐fully reject
Tired / exhausted	fully agree☐☐☐☐fully reject	fully agree☐☐☐☐fully reject	fully agree☐☐☐☐fully reject	fully agree☐☐☐☐fully reject
Comfortable in his / her body	fully agree☐☐☐☐fully reject	fully agree☐☐☐☐fully reject	fully agree☐☐☐☐fully reject	fully agree☐☐☐☐fully reject
Physically exhausted	fully agree☐☐☐☐fully reject	fully agree☐☐☐☐fully reject	fully agree☐☐☐☐fully reject	fully agree☐☐☐☐fully reject
Limited by physical complaints	fully agree☐☐☐☐fully reject	fully agree☐☐☐☐fully reject	fully agree☐☐☐☐fully reject	fully agree☐☐☐☐fully reject
Athletic / fit	fully agree☐☐☐☐fully reject	fully agree☐☐☐☐fully reject	fully agree☐☐☐☐fully reject	fully agree☐☐☐☐fully reject
Happy / good mood	fully agree☐☐☐☐fully reject	fully agree☐☐☐☐fully reject	fully agree☐☐☐☐fully reject	fully agree☐☐☐☐fully reject
Insecure / anxious	fully agree☐☐☐☐fully reject	fully agree☐☐☐☐fully reject	fully agree☐☐☐☐fully reject	fully agree☐☐☐☐fully reject
Annoyed / angry	fully agree☐☐☐☐fully reject	fully agree☐☐☐☐fully reject	fully agree☐☐☐☐fully reject	fully agree☐☐☐☐fully reject
Unhappy	fully agree☐☐☐☐fully reject	fully agree☐☐☐☐fully reject	fully agree☐☐☐☐fully reject	fully agree☐☐☐☐fully reject
Balanced	fully agree☐☐☐☐fully reject	fully agree☐☐☐☐fully reject	fully agree☐☐☐☐fully reject	fully agree☐☐☐☐fully reject
Self confident / self-assured	fully agree☐☐☐☐fully reject	fully agree☐☐☐☐fully reject	fully agree☐☐☐☐fully reject	fully agree☐☐☐☐fully reject
Stressed / nervous	fully agree☐☐☐☐fully reject	fully agree☐☐☐☐fully reject	fully agree☐☐☐☐fully reject	fully agree☐☐☐☐fully reject
